# Supplementary material for: Predicting in-hospital outcomes of patients with acute kidney injury
Source: Nat Commun. 2023 Jun 22;14:3739. doi: 10.1038/s41467-023-39474-6 (PMC10287760; doi:10.1038/s41467-023-39474-6)
Supplement: Supplementary file 1 — Supplementary Information [file 41467_2023_39474_MOESM1_ESM.pdf]

Supplemental Materials

Predicting in-hospital outcomes of patients with acute kidney injury

Changwei Wu [1], Yun Zhang [2], Sheng Nie [3], Daqing Hong [1], Jiajing Zhu [2], Zhi Chen [2], Bicheng Liu [4], Huafeng Liu [5], Qiongqiong Yang [6], Hua Li [7], Gang Xu [8], Jianping Weng [9], Yaozhong Kong [10], Qijun Wan [11], Yan Zha [12], Chunbo Chen [13], Hong Xu [14], Ying Hu [15], Yongjun Shi [16], Yilun Zhou [17] Guobin Su [18], Ying Tang [19], Mengchun Gong [20,21], Li Wang [1], Fanfan Hou [3, #], Yongguo Liu [2, #], Guisen Li [1, #]

Supplemental methods

Details of the prediction model

AKIEPM contains three parts: embedding, feature extraction, and output layers, whose framework is shown in Figure S1.

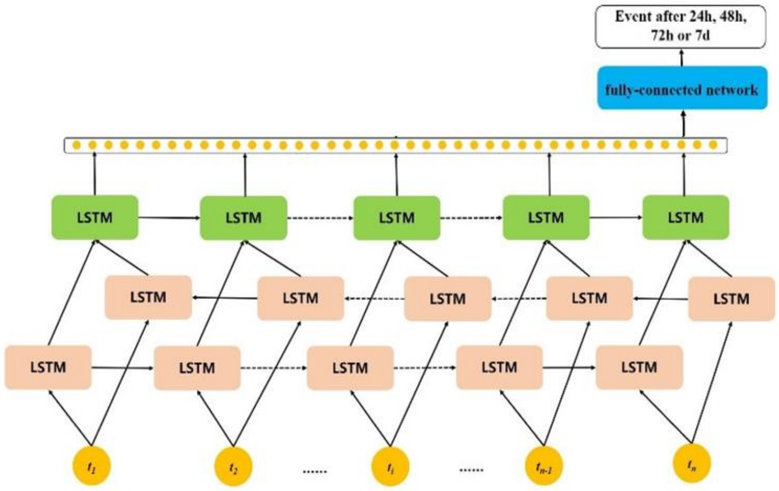

Figure S1 Framework of the AKI event prediction model AKIEPM.

(1) Embedding layer

The clinical variables include time, category, and value. Let $\mathbf{t}_\mathbf{t}^\mathbf{p} = \{s_1, x_t^1, \ldots, s_i, x_t^i, \ldots, s_V, x_t^V\}$, where s_i and x_t^i are the category and value of the *i*-th variable at time *t*, respectively. To help AKIEPM capture the dependence across different variables, we embedded the values of clinical variables into continuous vector $\mathbf{E}(\mathbf{t}_\mathbf{t}^\mathbf{p})$[23].

(2) Feature extraction layer

This layer is composed of a bidirectional LSTM layer and a one-way LSTM layer. The first layer employs 2000 bidirectional LSTM layers to extract the features from the forward and reverse of input sequence $\mathbf{x_t} = \mathbf{E}(\mathbf{t_t^p})$. The bidirectional LSTM is used to extract the hidden features for the embedding of sequential representation based on input, forget, and output units $\mathbf{i_t}, \mathbf{f_t}, \mathbf{o_t}$ of LSTM at time *t* with

$$\mathbf{i_t} = \mathrm{f}(\mathbf{W_{ie}}\mathbf{x_t} + \mathbf{W_{ih}}\mathbf{h_{t-1}} + w_{ic} \circ \mathbf{c_{t-1}} + b_i),$$

$$\mathbf{f_t} = \mathrm{f}\left(\mathbf{W_{fe}}\mathbf{x_t} + \mathbf{W_{fh}}\mathbf{h_{t-1}} + w_{fc} \circ \mathbf{c_{t-1}} + b_f\right),$$

$$\mathbf{c_t} = \mathbf{f_t} \circ \mathbf{c_{t-1}} + \mathbf{i_t} \circ \mathrm{g}(\mathbf{W_{ce}}\mathbf{x_t} + \mathbf{W_{ch}}\mathbf{h_{t-1}} + b_c),$$

$$\mathbf{o_t} = \mathrm{f}(\mathbf{W_{oe}}\mathbf{x_t} + \mathbf{W_{oh}}\mathbf{h_{t-1}} + w_{oc} \circ \mathbf{c_t} + b_o),$$

$$\mathbf{h_t} = \mathbf{o_t} \circ \mathrm{g}(\mathbf{c_i}).$$

Here, $\mathbf{c_t}$ is cell state, $\mathbf{x_t}$ and $\mathbf{h_t}$ are the input and hidden representations, respectively. f() adopts sigmoid function and g() adopts tanh nonlinear function with weight $\mathbf{W_{ie}}, \mathbf{W_{fe}}, \mathbf{W_{ce}}, \mathbf{W_{oe}}, \mathbf{W_{ih}}, \mathbf{W_{fh}}, \mathbf{W_{ch}}$ and $\mathbf{W_{oh}}$ for different inputs and gates with memory cells and outputs, as well as biases b_i, b_f, b_c and b_o. Cell state $\mathbf{c_t}$ is updated with a fraction of previous cell state $\mathbf{c_{t-1}}$ controlled by $\mathbf{f_t}$, and a new input state is created from the element-wise product of $\mathbf{i_t}$ and the output of the cell state by g() to remember the related features of death risk of AKI inpatients from historical records and current state. The peephole connection weights w_{ic}, w_{fc} and w_{oc} further influence input, forget, and output units.

The next one-way LSTM layer fuses the bidirectional output and obtains the vector representation $\mathbf{h_t}$ of all variables with attention mechanism at each moment in feature space, then the hidden representation of $\mathbf{t_t^p}$ can be defined as

$$\mathbf{H_t} = [\mathbf{h_1}, \ldots, \mathbf{h_t}, \ldots, \mathbf{h_T}].$$

(3) Output layer

Hidden state $\mathbf{H}$ is fed through the *softmax* layer to predict the events in 24h, 48h, 72h, and 7d, respectively.

$$\hat{y}_p^t = softmax(\mathbf{W_s H_t} + b_s).$$

Objective function

Let θ be the set of parameters, prediction probability vector $\hat{y}_p^t$ can be denoted by model posterior distribution $p = (y_p|X(p);\ \theta)$. We used the cross-entropy between ground truth y_p and prediction probabilities $\hat{y}_p^t$ to calculate the loss as follows,

$$L(\theta) = \frac{1}{N}\sum_{p=1}^{N}\left(\left(y_p\right)^{\mathrm{T}}\log\left(\hat{y}_p^t\right) + \left(1 - y_p\right)^{\mathrm{T}}\log\left(1 - \hat{y}_p^t\right)\right).$$

The bidirectional LSTM units adopted in AKIEPM extract the features from the forward and reverse of the input sequence to analyze the dependence among the input sequence, the forward sequence, and the reverse sequence when they are available for training. For testing and validating the model, AKIEPM only uses the history information without future data to predict the event based on the last input sequence, which is suitable for the clinical scenario, as shown in the following Figure S1. For example, when training the model, for input sequence t_i, the model can use the forward sequence t_{i-1} and the reverse sequence t_{i+1} to analyze their relevance; for input sequence t_n, the model uses the forward sequence t_{n-1} but not the reverse sequence. When testing and validating the model, if we input t_1 sequence, AKIEPM predicts the event after 24h, 48h, 72h, and 7d relative to time t_1; if we input the t_1, t_2, t_3 sequence, AKIEPM extracts the relevance among t_1, t_2, t_3 sequence and predicts the event after 24h, 48h, 72h and 7d relative to the last time t_3, which does not use the future data.

Supplemental Tables

Table S1 Demographic and baseline characteristics of the derivation, internal validation, and external validation cohorts

	Derivation cohort	Internal validation cohort	External validation cohort	*P* value
No. of patients	95257	27217	14610	
Female (%)	38656 (40.58)	10970 (40.31)	5755 (39.39)	0.023
Age (years)	62.1(48.7, 73.8)	62.1(48.5, 74.1)	65.1 (52.3, 76.8)	8.53E-90
SBP (mmHg)	121 (107, 137)	121 (107, 137)	124 (109, 140)	3.26E-30
DBP (mmHg)	72 (62, 81)	72 (62, 81)	72 (63, 82)	1.35E-06
MAP (mmHg)	88.7 (78.33, 99.00)	88.67 (78.67, 99.00)	90 (79.67, 101.00)	5.19E-17
Laboratory data				
SCr (μmol/L)	126.00 (92.00, 181.00)	126.80 (92.00, 183.00)	139.00 (105.25, 196.00)	2.58E-124
BUN (mmol/L)	9.00 (5.76, 14.04)	9.00 (5.80, 14.30)	9.93 (6.68, 14.84)	2.86E-68
BUN/SCr	15.00 (7.60, 60.85)	15.00 (8.00, 60.44)	64.13 (46.70, 94.19)	6.24E-270
Cystatin C (mg/L)	1.38 (0.95, 2.12)	1.38 (0.96, 2.13)	1.70 (1.19, 2.34)	1.45E-32
eGFR (ml/min per 1.73m^2)	47.09 (30.05, 70.07)	46.86 (29.69, 70.16)	40.84 (26.78, 57.61)	2.89E-155
Proteinuria (g/24h)	1.62 (0.43, 4.62)	1.66 (0.47, 4.52)	2.01 (0.49, 4.60)	0.297
GLU (mmol/L)	6.80 (5.30, 9.36)	6.80 (5.30, 9.35)	6.95 (5.49, 9.48)	6.08E-11
ALB (g/L)	33.40 (28.60, 38.20)	33.40 (28.60, 38.10)	35.10 (30.10, 39.70)	1.24E-118
ALT (IU/L)	24.00 (14.00, 46.40)	24.00 (14.00, 47.00)	21.00 (13.00, 40.48)	1.32E-41
AST (IU/L)	31.00 (20.00, 64.00)	31.00 (20.00, 64.00)	27.00 (18.00, 57.00)	7.07E-43

Table S1(continued)

	Derivation cohort	Internal validation cohort	External validation cohort	*P* value
TBIL (μmol/L)	13.70 (8.80, 23.10)	13.70 (8.80, 23.30)	12.20 (7.80, 20.60)	6.65E-61
DBIL (μmol/L)	4.50 (2.70, 8.90)	4.50 (2.60, 8.80)	4.90 (3.00, 9.40)	1.13E-33
WBC (10^9/L)	10.20 (7.00, 14.70)	10.20 (7.02, 14.73)	10.90 (7.76, 15.22)	4.36E-43
HB (g/L)	110 (90, 129)	110 (89, 129)	110 (89, 130)	0.919
PLT (10^9/L)	174.00 (114.00, 241.00)	175.00 (116.00, 242.00)	207.00 (142.00, 278.00)	1.23E-254
NLR	7.85 (3.92, 15.17)	7.89 (3.94, 15.18)	8.04 (4.14, 15.15)	0.005
CRP (mg/L)	40.17 (8.90, 103.36)	39.80 (9.00, 102.12)	45.51 (10.00, 123.00)	0.122
ESR (mm/h)	29.00 (12.00, 60.00)	29 (12.00, 61.25)	45 (21.00, 77.00)	1.23E-46
PCT (ng/mL)	0.76 (0.18, 4.60)	0.75 (0.17, 4.48)	0.65 (0.15, 4.55)	0.001
APTT (s)	35.20 (29.80, 41.70)	35.30 (29.80, 41.60)	31.50 (26.30, 39.10)	2.48E-305
D-dimer (μg/mL)	1.90 (0.63, 4.89)	1.93 (0.64, 4.86)	2.44 (0.96, 5.91)	5.89E-54
BNP (pg/mL)	196.00 (67.08, 648.03)	187.90 (67.35, 653.00)	191.50 (65.56, 669.37)	0.927
K (mmol/L)	4.06 (3.66, 4.51)	4.06 (3.66, 4.51)	4.07 (3.655, 4.53)	0.941
Na (mmol/L)	139.20 (136.00, 143.00)	139.10 (136.00, 142.90)	139.00 (135.60, 142.32)	3.64E-10
Ca (mmol/L)	1.12 (1.05, 1.19)	1.12 (1.04, 1.20)	1.10 (1.04, 1.15)	6.62E-61
Cl (mmol/L)	104.00 (99.80, 108.00)	104.00 (99.70, 108.00)	102.40 (98.30, 106.40)	1.48E-151
P (mmol/L)	1.16 (0.93, 1.42)	1.16 (0.93, 1.42)	1.15 (0.93, 1.40)	0.039

Table S1(continued)

	Derivation cohort	Internal validation cohort	External validation cohort	*P* value
Proteinuria (%)				1.23E-30
-	30289 (51.36)	8705 (51.62)	5293 (51.01)	
±	6572 (11.14)	1785 (10.58)	1010 (9.73)	
+	11716 (19.87)	3329 (19.74)	1821 (17.55)	
++	6031 (10.23)	1779 (10.55)	1462 (14.09)	
+++	3810 (6.46)	1106 (6.56)	695 (6.70)	
++++	555 (0.94)	160 (0.95)	95 (0.92)	
AKI stage (%)				3.66E-72
0	37478 (39.34)	10835 (39.81)	6906 (47.27)	
1	38417 (40.33)	10897 (40.04)	4952 (33.89)	
2	11264 (11.82)	3177 (11.67)	1579 (10.81)	
3	8098 (8.50)	2308 (8.48)	1173 (8.03)	
Baseline SCr (μmol/L) *	71.00 (52.00, 98.00)	71.00 (52.00, 98.67)	78.00 (59.53, 105.78)	5.39E-127
Comorbidities				
Diabetes (%)	17465 (18.33)	5136 (18.87)	3844 (26.31)	5.87E-114
Hypertension (%)	42089 (44.18)	12037 (44.23)	9246 (63.30)	0
Charlson comorbidity score	4 (2, 6)	4 (2, 6)	5 (3, 7)	0

Table S1(continued)

	Derivation cohort	Internal validation cohort	External validation cohort	*P* value
Surgery				
Major surgery (%)	9802 (10.29)	2712 (9.96)	317 (2.17)	1.09E-217
Cardiac surgery (%)	7391 (75.40)	2073 (76.44)	275 (86.75)	1.54E-05
Death (%)	1105 (1.16)	304 (1.12)	455 (3.11)	2.72E-82
Dialysis (%)	7654 (8.03)	2136 (7.85)	1415 (9.69)	9.54E-12
Length of hospitalization (day)	15.00 (9.00, 26.00)	15.00 (9.00, 26.00)	13.00 (8.00, 22.95)	3.38E-71
ICU stay (%)	5686 (5.97)	1600 (5.88)	675 (4.62)	6.04E-10
Length of ICU stay (h)	264.00 (120.00, 528.00)	263.41 (120.00, 512.86)	168.00 (48.00, 397.34)	1.16E-21
Mechanical ventilation (%)	16361 (17.18)	4685 (17.21)	4051 (27.73)	2.08E-211
Length of mechanical ventilation (h)	146.55 (30.73, 336.00)	145.02 (31.00, 352.52)	37.72 (18.52, 109.48)	5.25E-281

* The baseline SCr value in AKI patients is defined as the value at the time of AKI diagnosis established in this study.

Non-normally distributed variables were expressed as median and interquartile range and compared using the Kruskal–Wallis H test.

Abbreviations: ALB: albumin; ALT: alanine transaminase; APTT: activated partial thromboplastin time; AST: aspartate aminotransferase; BNP: brain natriuretic peptide; BUN: blood urea nitrogen; Ca: calcium; Cl: chloride; CRP: C-reactive protein; DBIL: direct bilirubin; DBP: diastolic blood pressure; eGFR: estimated glomerular filtration rate; ESR: erythrocyte sedimentation rate; GLU: glucose; HB: hemoglobin; ICU: intensive care units; K: potassium; MAP: mean arterial pressure; Na: sodium; NLR: neutrophil-to-lymphocyte ratio; PCT: procalcitonin; P: phosphorus, PLT: platelet; SBP: systolic blood pressure; SCr: serum creatinine; TBIL: total bilirubin; UA: uric acid; WBC: white blood cell.

Table S2 Demographic and baseline characteristics of death and survival cohorts

	Survival patients	Dead patients	*P* value
No. of patients	135220	1864	
Female (%)	54781 (40.51)	600 (32.19)	1.85E-13
Age (years)	62.5(49.0, 74.1)	67.4 (51.5, 80.1)	3.46E-28
SBP (mmHg)	122 (108, 138)	110 (94, 127)	3.25E-98
DBP (mmHg)	72 (63, 82)	62 (52, 72)	6.74E-165
MAP (mmHg)	89.33 (79.00, 99.67)	78.00 (67.33, 89.00)	8.22E-154
Laboratory data			
SCr (μmol/L)	127.30 (93.00, 182.54)	156.00 (108.85, 224.00)	2.16E-46
BUN (mmol/L)	9.07 (5.81, 14.10)	12.70 (8.38, 19.00)	3.66E-101
BUN/SCr	15.53 (8.00, 62.46)	15.00 (9.00, 29.63)	0.321
Cystatin C (mg/L)	1.38 (0.96, 2.12)	1.90 (1.26, 2.82)	6.38E-29
eGFR (ml/min per 1.73m^2)	46.38 (29.70, 68.84)	35.97 (23.08, 55.30)	4.31E-49
Proteinuria (g/24h)	1.65 (0.45, 4.61)	0.57 (0.34, 1.96)	0.120
GLU (mmol/L)	6.80 (5.31, 9.32)	8.64 (6.28, 12.00)	1.90E-72
ALB (g/L)	33.60 (28.80, 38.30)	31.00 (26.30, 35.80)	1.22E-44
ALT (IU/L)	23.00 (14.00, 46.00)	35.05 (18.00, 95.75)	5.10E-59
AST (IU/L)	30.00 (19.50, 62.00)	60.00 (30.00, 174.80)	7.82E-133
TBIL (μmol/L)	13.50 (8.70, 22.80)	16.60 (10.10, 31.40)	7.43E-28

Table S2 (continued)

	Survival patients	Dead patients	*P* value
DBIL (μmol/L)	4.53 (2.70, 8.80)	7.50 (4.10, 16.80)	6.93E-82
WBC (10×10^9/L)	10.27 (7.10, 14.70)	13.36 (8.88, 18.75)	3.48E-64
NLR	7.84 (3.93, 15.11)	10.76 (5.98, 19.45)	5.17E-42
PLT ($\times10^9$/L)	178.00 (118.00, 246.00)	134.00 (72.00, 212.00)	2.78E-66
HB (g/L)	110.00 (90.00, 129.00)	101.00 (79.00, 124.00)	1.40E-28
CRP (mg/L)	39.70 (8.81, 102.76)	79.64 (25.59, 148.15)	1.39E-26
ESR (mm/h)	30.00 (12.00, 62.00)	32.50 (14.75, 62.00)	0.567
PCT (ng/mL)	0.72 (0.17, 4.45)	2.28 (0.46, 11.77)	9.43E-53
APTT (s)	34.90 (29.30, 41.30)	40.55 (33.90, 50.15)	6.83E-108
D-dimer (μg/mL)	1.92 (0.65, 4.87)	5.60 (2.46, 14.49)	7.15E-181
BNP (pg/mL)	191.90 (66.35, 639.25)	444.40 (126.50, 1588.80)	1.77E-14
K (mmol/L)	4.06 (3.66, 4.51)	4.19 (3.66, 4.84)	1.15E-11
Na (mmol/L)	139.10 (136.00, 142.80)	142.00 (136.50, 150.00)	1.28E-65
Ca (mmol/L)	1.11 (1.04, 1.18)	1.06 (0.98, 1.14)	1.32E-24
Cl (mmol/L)	103.90 (99.60, 108.00)	103.80 (98.00, 111.30)	0.005
P (mmol/L)	1.16 (0.93, 1.42)	1.15 (0.82, 1.69)	0.242

Table S2 (continued)

	Survival patients	Dead patients	*P* value
Proteinuria (%)			3.59E-15
-	43867 (51.53)	420 (38.96)	
±	9228 (10.84)	139 (12.89)	
+	16600 (19.50)	266 (24.68)	
++	9104 (10.69)	168 (15.58)	
+++	5533 (6.50)	78 (7.24)	
++++	803 (0.94)	7 (0.65)	
AKI stage (%)			3.96E-229
0	54645 (40.41)	574 (30.79)	
1	53852 (39.83)	414 (22.21)	
2	15644 (11.57)	376 (20.17)	
3	11079 (8.19)	500 (26.82)	
Baseline SCr (μmol/L) *	71.75 (52.50, 99.00)	86.50 (63.00, 127.80)	3.64E-56
Comorbidities			
Diabetes (%)	26033 (19.255)	412 (22.10)	0.002
Hypertension (%)	62332 (46.10)	1040 (55.79)	1.01E-16
Charlson comorbidity score	4.00 (3, 6)	5.00 (3, 7)	2.20E-56

Table S2 (continued)

	Survival patients	Dead patients	*P* value
Surgery			
Major surgery (%)	12761 (9.44)	70 (3.76)	9.99E-21
Cardiac surgery (%)	9685 (75.90)	54 (77.14)	0.889
Dialysis (%)	10743 (7.94)	462 (24.79)	0.002
Length of hospitalization (day)	15.00 (9.00, 26.00)	10.57 (4.28, 22.95)	3.99E-58
ICU stay (%)	7666 (5.67)	295 (15.83)	5.42E-55
Length of ICU stay (h)	258.34 (119.85, 528.00)	183.17 (81.18, 396.74)	2.31E-04
Mechanical ventilation (%)	23930 (17.70)	1167 (62.61)	0
Length of mechanical ventilation (h)	120.48 (24.01, 312.36)	66.58 (22.39, 201.54)	2.90E-15

[*] The baseline SCr value in AKI patients is defined as the value at the time of AKI diagnosis established in this study.

Non-normally distributed variables were expressed as median and interquartile range and compared using the Kruskal–Wallis H test.

Abbreviations: ALB: albumin; ALT: alanine transaminase; APTT: activated partial thromboplastin time; AST: aspartate aminotransferase; BNP: brain natriuretic peptide; BUN: blood urea nitrogen; Ca: calcium; Cl: chloride; CRP: C-reactive protein; DBIL: direct bilirubin; DBP: diastolic blood pressure; eGFR: estimated glomerular filtration rate; ESR: erythrocyte sedimentation rate; GLU: glucose; HB: hemoglobin; ICU: intensive care units; K: potassium; MAP: mean arterial pressure; Na: sodium; NLR: neutrophil-to-lymphocyte ratio; PCT: procalcitonin; P: phosphorus, PLT: platelet; SBP: systolic blood pressure; SCr: serum creatinine; TBIL: total bilirubin; UA: uric acid; WBC: white blood cell.

Table S3 Various evaluation indicators of the comparing algorithmic models.

	Bilstm				BiSinglelistm			
	Outcome of death		Outcome of dialysis		Outcome of death		Outcome of dialysis	
	F-score	AUROC	F-score	AUROC	F-score	AUROC	F-score	AUROC
Derivation (%)								
24h	58.12	62.40	59.86	63.98	58.07	60.50	58.63	64.46
48h	53.80	58.19	57.38	63.31	44.95	59.79	56.50	63.82
72h	55.66	61.21	57.56	61.38	57.11	59.97	56.38	63.42
7d	54.34	57.26	57.55	60.89	56.73	60.01	55.41	60.79
Internal Validation (%)								
24h	57.38	51.79	60.23	64.17	56.15	57.41	58.70	64.71
48h	50.91	53.71	56.79	63.18	36.82	56.19	56.27	63.92
72h	52.98	57.87	57.99	61.95	53.58	54.86	55.95	63.32
7d	53.92	55.51	57.57	61.09	56.06	59.21	56.06	61.20
External Validation (%)								
24h	4.41	63.05	43.58	51.07	39.00	38.12	42.87	52.62
48h	36.02	37.63	14.27	51.56	34.53	42.99	37.48	51.47
72h	12.04	32.58	38.69	49.04	38.60	35.92	36.24	51.42
7d	51.60	56.24	39.64	49.29	53.74	52.18	31.88	49.95

Table S4 Index included in predictive mode

	index
Demographic characteristics	Age, gender
Clinical characteristics	MAP, SBP, DBP, AKI stage, comorbidities
Operation	ICU, mechanical ventilation, surgery, length of ICU, and mechanical ventilation
Treatment	drug
Experiment characteristics	ALB, ALT, APTT, AST, BUN, BNP, BUN/SCr, Ca, Cl, CRP, cTn, Cys-C, DBIL, D-Dimer, eGFR, ESR, FDP, FIB, Glu, HB, INR, LDH, NLR, Na, P, PCT, PLT, proteinuria, 24 proteinuria, PT, RBC, SCr, TBIL, TC, TCa, TG, TP, TT, UA, WBC, Blood gas analysis factors.

Abbreviations:

AKI: acute kidney injury; ALB: albumin; ALT: alanine transaminase; APTT: activated partial thromboplastin time; AST: aspartate aminotransferase; BUN: blood urea nitrogen; BNP: brain natriuretic peptide; Ca: calcium; Cl: chloride; CRP: C-reactive protein; cTn: cardiac troponin; DBIL: direct bilirubin; DBP: diastolic blood pressure; eGFR: estimated glomerular filtration rate; ESR: erythrocyte sedimentation rate; FDP: Fibrinogen and Fibrin Degradation Products; FIB: fibrinogen; Glu: glucose; HB: hemoglobin; ICU: intensive care units; INR: international normalized ratio; K: kalium; LDH: lactate dehydrogenase; MAP: mean arterial pressure; NLR: neutrophil-to-lymphocyte ratio; Na: sodium; P: phosphorus; PCT: procalcitonin; PLT: platelet; PT: prothrombin time; RBC: red blood cell; SBP: systolic blood pressure; SCr: serum creatinine; TBIL: total bilirubin; TC: total cholesterol; TCa: total calcium; TG: Triglyceride; TP: total protein; TT: thrombin time; UA: uric acid; WBC: white blood cell.

Blood gas analysis factors included pH, ab-hco3, BE, bevt, hco3, sbe, sb-hco3, tco2.

Standard Tables in CRDS

Patient and Visit Information

Table S5 Patient information.

Column name	Type of Variables	Required Field	Note
person_id	string	Required	Unique identification of the patient (patient master index number)
person_name	string	De-sensitization	Name
gender	string	Required	Gender of the patient (male or female)
birthday	date	Required	Date of birth（YYYY-MM-DD）
race	string	Required	Ethnic
location	string	De-sensitization	Patient Address (Geolocation)
Intersecting_nation	string	Required	Origin
card_id	string	De-sensitization	ID number

Table S6 Visit Information

Column name	Type of Variables	Required Field	Note
visit_record_id	string	Required	Unique identification of the current record, visit number
person_id	string	Required	From the im_person table, the same method for obtaining person-id as this table
visit_type	string	Required	Emergency, outpatient, inpatient, medical examination, etc
visit_start_date	date	Required	Date of admission/visit
visit_end_date	date	Required	Date of discharge
department_id	string	Required	Admission Department
dept_discharge_from	string	Required	Discharge department
out_condition	string	Required	Discharge status(eg. cured, improved, worsened)
leave_mode	string	Required	Mode of discharge (Medical advice for discharge, transfer to a superior or subordinate hospital for further treatment, death, etc.)
total_cost	number	Required	Total cost during hospitalization

Diagnosis and treatment information

Table S7 Diagnosis information

Column name	Type of Variables	Required Field	Note
person_id	string	Required	Patient ID, from the im_person table
visit_record_id	string	Required	Visit record ID, from the im_visit table
condition_code	string	Required	Diagnostic code, (eg. ICD10 code)
condition_name	string	Required	Diagnostic name
condition_start_date	Timestamp	Required	Diagnostic time
condition_type	string	Required	Outpatient diagnosis, admission diagnosis, discharge diagnosis, etc
diagonois_type	string	Required	Type of diagnosis (major diagnosis, secondary diagnosis)

Table S8 Prescription Information

Column name	Type of Variables	Required Field	Note
person_id	string	Required	Patient ID, from the im_person table
visit_record_id	string	Required	Visit record ID, from the im_visit table
prescription_index	string	Required	Prescription attributes (long-term, temporary, etc.)
prescription_type	string	Required	Types of Prescription (medication, procedures, care, testing, etc.)
prescription_name	string	Required	Prescription name
prescription_start_date	Timestamp	Required	Start time of prescription
prescription_end_date	Timestamp	Required	End time of prescription
drug_type	string	Required	Drug type (such as western medicine, Chinese herbal medicine, Chinese patent medicine, etc.)
drug_frequency	string	Required	Frequency of medication (such as qd)
drug_method	string	Required	Mode of administration, such as oral, intravenous, topical
drug_dose	Integer	Required	Dose, such as 50
drug_dose_unit	string	Required	Dose unit, such as mg

Table S9 Laboratory Testing

Column name	Type of Variables	Required Field	Note
person_id	string	Required	Patient ID, from the im_person table
visit_record_id	string	Required	Visit record ID, from the im_visit table
measurement_name	string	Required	Inspection sub-item name
value_as_number	float	Required	Inspection numerical results
value_as_category	string	Required	Inspection non numerical results
category_of_reference	string	Required	Reference value for non numerical test results
unit	string	Required	Units of numerical results
range_low	float	Required	Lower limit of reference value for numerical results
range_high	float	Required	Upper limit of reference value for numerical results
group_measurement_name	string	Required	Inspection large item name
apply_time	Timestamp	Required	Application time: including hours, minutes, and seconds (LIS)
collection_ time	Timestamp	Required	Sample collection time: including hours, minutes, and seconds (LIS)
receiving_ time	Timestamp	Required	Sample acceptance time: including hours, minutes, and seconds (LIS)
measurement_time	Timestamp	Required	Sample detection time: including hours, minutes, and seconds (LIS)
report_time	Timestamp	Required	Report time: including hours, minutes, and seconds (LIS)
sample_name	string	Required	Sample name (such as serum, plasma, urine)

Table S10 Surgery information

Column name	Type of Variables	Required Field	Note
person_id	string	Required	Patient ID, from the im_person table
visit_record_id	string	Required	Visit record ID, from the im_visit table
procedure_name	string	Required	Surgical name
procedure_code	string	Required	Codes of surgical procedures (such as ICD9-CM)
procedure_date	Timestamp	Required	Surgical date and time
operation_grade	string	Required	Surgical grade
anesthesia_method	string	Required	Name of anesthesia method
anesthesia_level	string	Required	Anesthesia level (ASA level)
incision_healing	string	Required	Incision healing level

Table S11 Medical Imaging Examination

Column name	Type of Variables	Required Field	Note
person_id	string	Required	Patient ID, from the im_person table
visit_record_id	string	Required	Visit record ID, from the im_visit table
technology_name	string	Required	Inspection name
technology_type	string	Required	Inspection category (CT, B-ultrasound, MRI...)
technology_apply_date	Timestamp	Required	Application time, including hours, minutes, and seconds
technology_report_time	Timestamp	Required	Report time, including hours, minutes, and seconds
position	string	Required	Inspection site
check_result	string	Required	Description of inspection results
check_doctor_desc	string	Required	Physician's diagnostic opinion

Table S12 Death Records

Column name	Type of Variables	Required Field	Note
person_id	string	Required	Patient ID, from the im_person table
visit_record_id	string	Required	Visit record ID, from the im_visit table
death_name	string	Required	Death record name
death_date	date	Required	Death record date
death_reason	string	Required	Direct cause of death
death_condition_name	string	Required	Death diagnosis name
death_condition_code	string	Required	Death diagnostic codes (such as ICD-10)

Medical records information

Table S13 Medical Records

Column name	Type of Variables	Required Field	Note
person_id	string	Required	Patient ID, from the im_person table
visit_record_id	string	Required	Visit record ID, from the im_visit table
note_date	Timestamp	Required	Text record time
note_type	string	Required	Text type (admission records, course records, discharge summaries, etc.)
note_text	CLOB	De-sensitization	Text content

Table S14 Vital Signs

Column name	Type of Variables	Required Field	Note
person_id	string	Required	Patient ID, from the im_person table
visit_record_id	string	Required	Visit record ID, from the im_visit table
measure_time	Timestamp	Required	Measurement time (accurate to hours, minutes, seconds)
entry_name	string	Required	Project name (temperature, pulse, respiration, blood pressure, height, weight, etc.)
measured_value	string	Required	Measurement value
measured_unit	string	Required	Unit
reference_value	string	Required	Reference value
