# Supplementary material for: Predicting in-hospital outcomes of patients with acute kidney injury
Source: Nat Commun. 2023 Jun 22;14:3739. doi: 10.1038/s41467-023-39474-6 (PMC10287760; doi:10.1038/s41467-023-39474-6)
Supplement: Supplementary file 3 — Reporting Summary [file 41467_2023_39474_MOESM3_ESM.pdf]

nature portfolio

Corresponding author(s): Guisen Li, Yongguo Liu, Fanfan Hou

Last updated by author(s): May 31, 2023

Reporting Summary

Nature Portfolio wishes to improve the reproducibility of the work that we publish. This form provides structure for consistency and transparency in reporting. For further information on Nature Portfolio policies, see our Editorial Policies and the Editorial Policy Checklist.

Statistics

For all statistical analyses, confirm that the following items are present in the figure legend, table legend, main text, or Methods section.

n/a	Confirmed	
[ ]	[x]	The exact sample size (n) for each experimental group/condition, given as a discrete number and unit of measurement
[ ]	[x]	A statement on whether measurements were taken from distinct samples or whether the same sample was measured repeatedly
[ ]	[x]	The statistical test(s) used AND whether they are one- or two-sided<br>*Only common tests should be described solely by name; describe more complex techniques in the Methods section.*
[ ]	[x]	A description of all covariates tested
[ ]	[x]	A description of any assumptions or corrections, such as tests of normality and adjustment for multiple comparisons
[ ]	[x]	A full description of the statistical parameters including central tendency (e.g. means) or other basic estimates (e.g. regression coefficient) AND variation (e.g. standard deviation) or associated estimates of uncertainty (e.g. confidence intervals)
[x]	[ ]	For null hypothesis testing, the test statistic (e.g. F, t, r) with confidence intervals, effect sizes, degrees of freedom and P value noted<br>*Give P values as exact values whenever suitable.*
[x]	[ ]	For Bayesian analysis, information on the choice of priors and Markov chain Monte Carlo settings
[ ]	[x]	For hierarchical and complex designs, identification of the appropriate level for tests and full reporting of outcomes
[x]	[ ]	Estimates of effect sizes (e.g. Cohen's d, Pearson's r), indicating how they were calculated

Our web collection on statistics for biologists contains articles on many of the points above.

Software and code

Policy information about availability of computer code

Data collection	Data was collected from each collaborating hospitals of the National Clinical Research Centre for Chronic Kidney Diseases (NCRCCKD) by professional Extract-Transform-Load (ETL) engineers according to the standard field forms (SOP for big data including visit, diagnosis, surgery, laboratory metrics, prescriptions, event, department, Imaging data, Vital signs, and Medical records). We used Oracle Database 12c Enterprise Edition Release 12.2.0.1.0 with SQL sentences to capture the raw data from the HIS, LIS, PACS and other systems of the collaborating hospitals.
Data analysis	The data analysis by AKIEPM was coded by TensorFlow 1.15 and Python 3.7 with patient data, whose code is available at https://github.com/yunzhangwww/AKIEPM.

For manuscripts utilizing custom algorithms or software that are central to the research but not yet described in published literature, software must be made available to editors and reviewers. We strongly encourage code deposition in a community repository (e.g. GitHub). See the Nature Portfolio guidelines for submitting code & software for further information.

Data

Policy information about availability of data

All manuscripts must include a data availability statement. This statement should provide the following information, where applicable:

- Accession codes, unique identifiers, or web links for publicly available datasets
- A description of any restrictions on data availability
- For clinical datasets or third party data, please ensure that the statement adheres to our policy

Data for figures are provided with this paper and are available at https://github.com/yunzhangwww/AKIEPM. In according to with current National Health Commission of China confidential policy, the raw data used in this study is only available for the researchers participating in this study. Thus, we are not allowed to distribute or make the data publicly available to other parties. CRDS data is currently only openly shared with participating collaborators. If you are willing to cooperate, please contact the corresponding author Guisen Li (guisenli@163.com), and we will respond within two weeks.

Human research participants

Policy information about studies involving human research participants and Sex and Gender in Research.

Reporting on sex and gender	In this study population, there were a total of 55381 women, accounting for 40.40% of the total population. In the testing group, internal validation group, and external validation group, women accounted for 40.58%, 40.31%, and 39.39%, respectively. In the death group and survival group, women accounted for 40.51% and 32.19%, respectively. In order to verify the predictive effect of the prediction model among different genders, we conducted subgroup analysis and the results showed that the model had good predictive performance in both female and male subgroups.
Population characteristics	Table 1 shows the baseline characteristics of the derivation, internal, and external validation cohorts. The external validation cohort had a higher percentage of patients with AKI stage 0, higher incidence of mortality, need for dialysis, use of mechanical ventilation, hypertension, and diabetes, higher Charlson comorbidity scores, and a lower percentage of patients who underwent major surgery and ICU admission than the other cohorts. For laboratory parameters, SCr, C-reactive protein, chloride, procalcitonin, and erythrocyte sedimentation rate were higher in the external validation group.<br><br>We compared the baseline characteristics between the death and survival cohorts (Table 2). Overall, 1,864 patients with AKI (1·38%) died in hospital. These dead patients had higher baseline SCr and proteinuria, as well as more severe AKI stage. The prevalence of hypertension and diabetes in the death group was higher than that in the survival group, and Charlson's complication score was also higher than that in the survival group. In the death group, more patients received dialysis or mechanical ventilation, and stayed in the ICU (Table 2).
Recruitment	The study population was derived from the Chinese Renal Disease Data System (CRDS), a large multicenter retrospective study cohort of 7,084,339 patients hospitalized at 19 medical centers throughout China from January 1, 2000, to May 26, 2021.
Ethics oversight	The study protocol was approved by the Medical Ethics Committee of Sichuan Provincial People's Hospital (approval number: 2022-83), and Nanfang Hospital, Southern Medical University (approval number: NFEC-2019-213), which waived the requirement for patient informed consent due to the retrospective nature of the study. This study was also approved by the China Office of Human Genetic Resources for Data Preservation Application (approval number: 2021-BC0037) and was performed by the Strengthening the Reporting of Observational Studies in Epidemiology (STROBE) guidelines.

Note that full information on the approval of the study protocol must also be provided in the manuscript.

Field-specific reporting

Please select the one below that is the best fit for your research. If you are not sure, read the appropriate sections before making your selection.

☒ Life sciences ☐ Behavioural & social sciences ☐ Ecological, evolutionary & environmental sciences

For a reference copy of the document with all sections, see nature.com/documents/nr-reporting-summary-flat.pdf

Life sciences study design

All studies must disclose on these points even when the disclosure is negative.

Sample size	No sample-size calculation was performed in this study. This study used deep learning method to construct a predictive model. A large sample size is beneficial for model construction and validation. Therefore, we included all patients metting including and excluding criteria of our study in the Chinese Renal Disease Data System,a total of 137084 patients.
Data exclusions	The exclusion criteria were as follows: (a) patients who had less than two serum creatinine (SCr) results during hospitalization; (b) patients <18 years old; (c) patients who had HIV; and (d) patients who had end-stage kidney disease (ESKD, defined as maintenance dialysis, kidney transplantation, or eGFR <15 ml/min per 1.73m2). Patients who had undergone dialysis prior to developing AKI were also excluded when

	dialysis was analyzed as an in-hospital outcome.
Replication	In this study, we train the deep learning model in derivation cohort and test in internal validation cohort with 100 epochs and validate in external validation cohort to obtain the final results.
Randomization	The study was based on real-world data for predictive model development, without intervention or clinical trials, involving no experimental or control groups.
Blinding	The study was based on real-world data for predictive model development, without intervention or clinical trials, involving no blinding.

Reporting for specific materials, systems and methods

We require information from authors about some types of materials, experimental systems and methods used in many studies. Here, indicate whether each material, system or method listed is relevant to your study. If you are not sure if a list item applies to your research, read the appropriate section before selecting a response.

Materials & experimental systems

n/a	Involved in the study	
☒	☐	Antibodies
☒	☐	Eukaryotic cell lines
☒	☐	Palaeontology and archaeology
☒	☐	Animals and other organisms
☐	☒	Clinical data
☒	☐	Dual use research of concern

Methods

n/a	Involved in the study	
☒	☐	ChIP-seq
☒	☐	Flow cytometry
☒	☐	MRI-based neuroimaging

Clinical data

Policy information about clinical studies

All manuscripts should comply with the ICMJEguidelines for publication of clinical research and a completedCONSORT checklist must be included with all submissions.

Clinical trial registration	This is a a retrospective study based on the Chinese Renal Disease Data System, not the interventional trial.
Study protocol	The study protocol was described in Methods of manuscript.
Data collection	The study population was derived from the Chinese Renal Disease Data System (CRDS), a large multicenter retrospective study cohort of 7,084,339 patients hospitalized at 19 medical centers throughout China from January 1, 2000, to May 26, 2021. The dataset consisted of information from hospital electronic health records in digital format. The number of independent entries in the dataset was approximately 2·8 billion, including 37,224 features. We extracted adult patients' data, including outpatient visits, admissions, diagnoses as International Statistical Classification of Diseases and Related Health Problems codes, surgical procedures (including date, names, and codes [ICD-9-CM-3]), vital signs, stay-in ICU, mechanical ventilation, laboratory results (including—but not limited to—biochemistry, hematology, cytology, microbiology, and histopathology), medications and prescriptions, orders, dialysis (including hemodialysis, peritoneal dialysis, continuous renal replacement therapy), and in-hospital death.<br><br>Patients who developed AKI during hospitalization (including community-acquired AKI) were selected for further screening. The exclusion criteria were as follows: (a) patients who had less than two serum creatinine (SCr) results during hospitalization; (b) patients <18 years old; (c) patients who had HIV; and (d) patients who had end-stage kidney disease (ESKD, defined as maintenance dialysis, kidney transplantation, or eGFR <15 ml/min per 1.73m2).
Outcomes	The primary and secondary outcomes were in-hospital death and the need for dialysis, respectively. In this study, dialysis included temporary or maintenance hemodialysis and peritoneal dialysis, and continuous renal replacement therapy (CRRT).
